# Secondary Osteosarcoma After Carbon‐Ion Radiotherapy for Desmoid‐Type Fibromatosis: A Case Report

**DOI:** 10.1002/cnr2.70062

**Published:** 2024-11-19

**Authors:** Mizuki Aketo, Makoto Emori, Kohichi Takada, Kazuyuki Murase, Yohei Arihara, Junya Shimizu, Yasutaka Murahashi, Masahiko Okamoto, Shintaro Sugita, Atsushi Teramoto

**Affiliations:** ^1^ Department of Orthopaedic Surgery Sapporo Medical University School of Medicine Sapporo Japan; ^2^ Department of Medical Oncology Sapporo Medical University School of Medicine Sapporo Japan; ^3^ Department of Radiation Oncology Gunma University Graduate School of Medicine Maebashi Japan; ^4^ Gunma University Heavy Ion Medical Center Maebashi Japan; ^5^ Department of Surgical Pathology Sapporo Medical University School of Medicine Sapporo Japan

**Keywords:** carbon‐ion radiotherapy, desmoid‐type fibromatosis, osteosarcoma, post‐irradiation sarcoma

## Abstract

**Background:**

Radiotherapy is considered an alternative treatment for unresectable or pharmacologically resistant desmoid‐type fibromatosis. While it results in relatively good local control, the risk of secondary malignancy remains a concern.

**Case:**

We present a case of secondary osteosarcoma after carbon‐ion radiation therapy (CIRT). A 31‐year‐old male patient presented with left thigh pain. The tumor was located between the left gluteus maximus and gluteus medius and extended to the vastus lateralis and biceps femoris. It was diagnosed as desmoid‐type fibromatosis after needle biopsy. The patient was treated with several medications, including a cyclooxygenase 2 inhibitor and tamoxifen; however, his left thigh pain did not improve. He was treated with CIRT 1 year after diagnosis (67.2 Gy [relative biological effectiveness] 16fr/4wks). He developed osteosarcoma of the left femur 8 years later. He underwent chemotherapy and tumor excision with disarticulation of the left hip. Pulmonary metastasis was detected 6 and 17 months after the definitive surgery and excised using metastasectomy. However, he died due to the recurrence of multiple pulmonary metastases 29 months after the definitive surgery.

**Conclusions:**

In this case, we believe that the low radiation dose to the femur may have caused secondary malignancy.

**Abbreviations**
CIRT: carbon ion radiotherapy
DF: desmoid‐type fibromatosis
MRI: magnetic resonance imaging
RT: radiotherapy

## 1 Introduction

Desmoid fibromatosis (DF) is defined as a clonal fibroblastic proliferation that occurs in deep soft tissues and is characterized by non‐metastatic infiltrative proliferation and a propensity for local recurrence [1]. DF can affect all body parts, including the extremities, trunk, and abdomen. Treatment is usually based on anatomical location and must be determined in stages and in consultation with the patient [2]. Initial observation (active surveillance) for asymptomatic lesions is an acceptable strategy. However, active treatment, including surgery, radiotherapy (RT), and systemic treatment, should be considered for cases of disease progression. Surgery is considered an option for DF if adequate resection can be performed without significant complications [2, 3].

RT is considered an alternative standard local therapy for DF when surgical resection is not possible due to location, tumor size, or potential functional impairment [4]. RT results in relatively good local control but the risk of secondary malignancies remains a concern. Here, we present a rare case of secondary osteosarcoma after carbon‐ion radiotherapy (CIRT) for DF. Although the patient achieved tumor control and pain relief after CIRT, secondary malignancy developed 8 years after the treatment. The low radiation dose to the femur may have caused secondary malignancy.

## 2 Case

**FIGURE 1:**
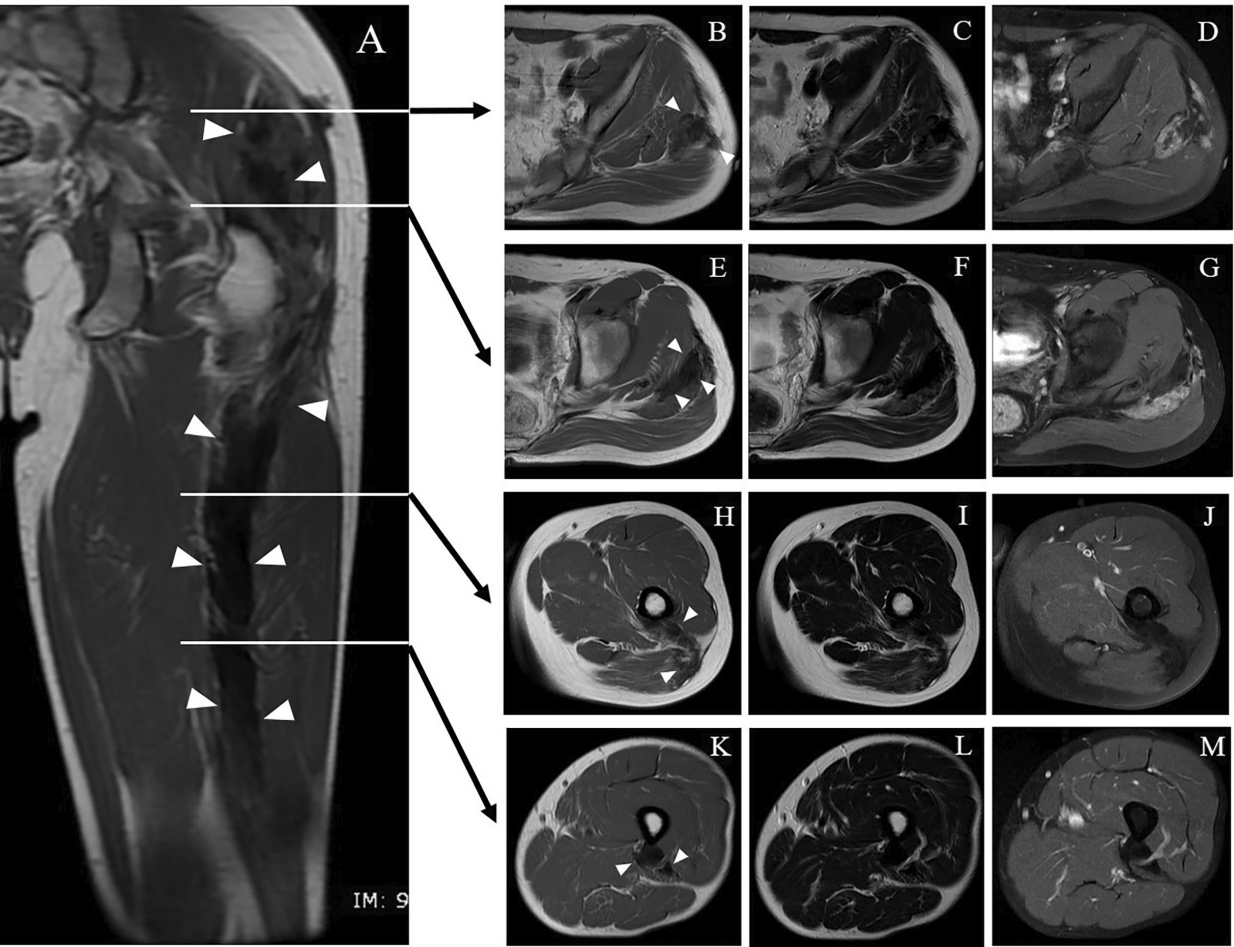
Thirty‐one‐year‐old male patient. Magnetic resonance images of the left hemipelvis show homogeneously low‐intense lesions on T1‐, T2‐, and Gd‐contrast enhancement fat suppression T1‐weighted images of the left gluteus maximus and gluteus medius muscles, extending to the vastus lateralis and biceps femoris. The maximum diameter of the lesion is 35 mm. The tumor is indicated by arrowheads. (A) T1WI coronal sequence. (B, E, H, K) T1WI axial. The tumor is shown using arrowheads. (C, F, I, L) T2WI axial sequence. (D, G, J, M) Gd‐contrast enhancement fat suppression T1WI.

**FIGURE 2:**
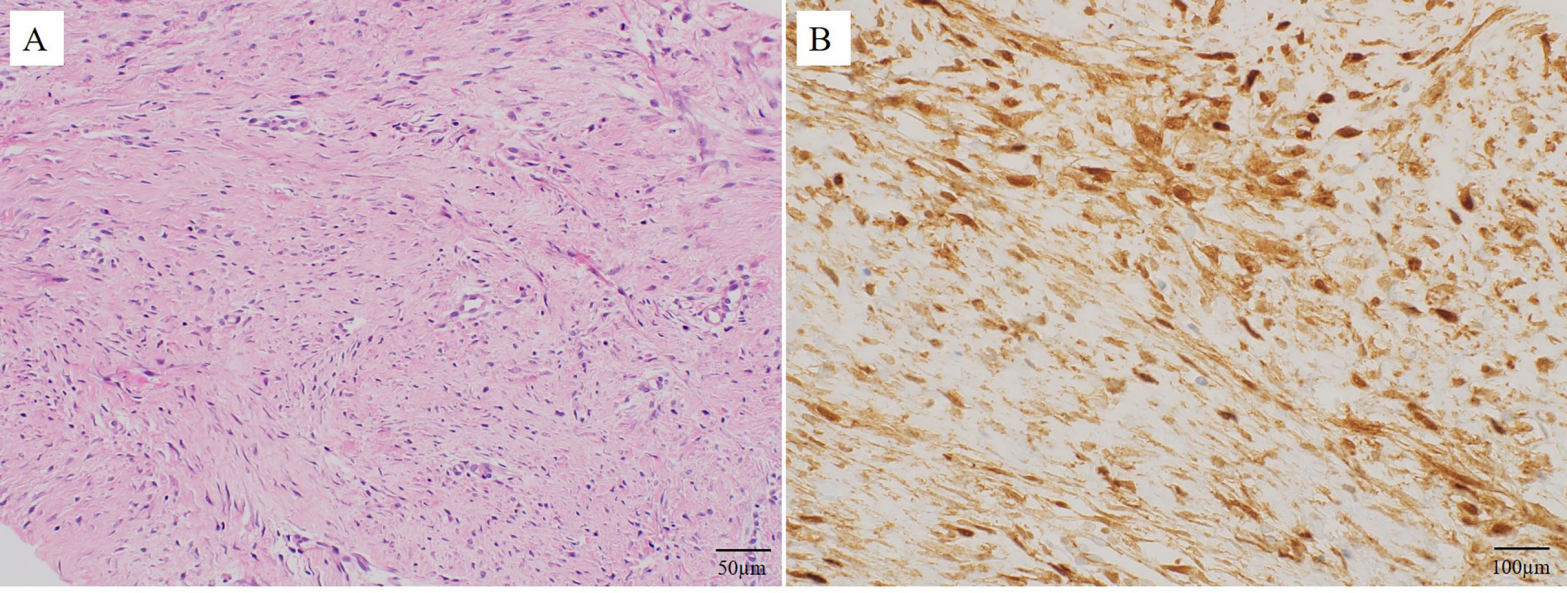
(A) Hematoxylin and eosin (HE) stain (original magnification ×200). The tumor shows proliferation of uniform‐appearing spindle‐shaped cells lacking cytological atypia. The tumor has small vessels and edema, and abundant fibrous matrix. Desmoid‐type fibromatosis was pathologically diagnosed. (B) Nuclear expression of β‐catenin detected by immunohistochemistry (original magnification ×400).

**FIGURE 3:**
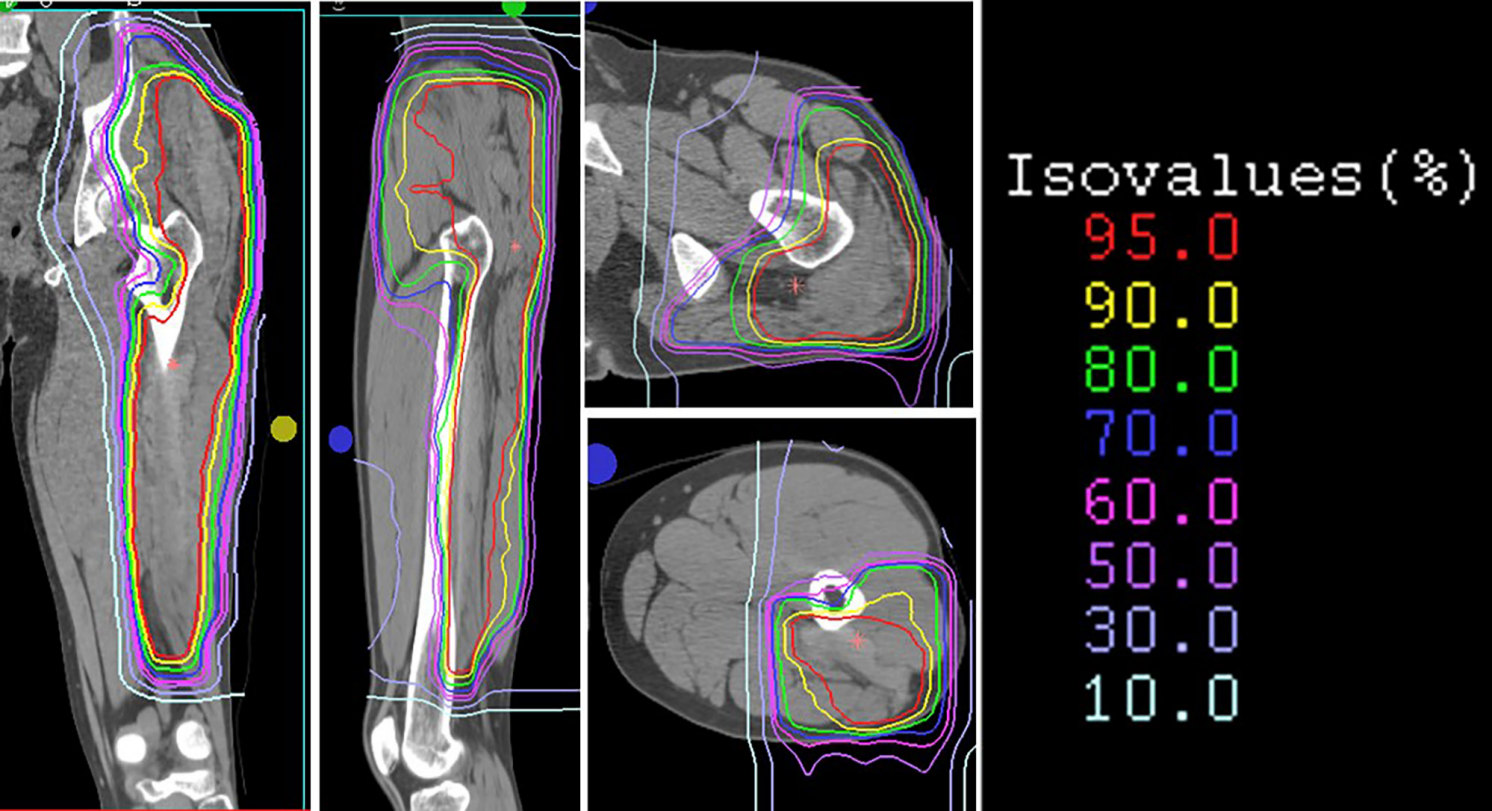
Carbon‐ion treatment plan for the patient. The multi‐linear circles show the dose distribution.

**FIGURE 4:**
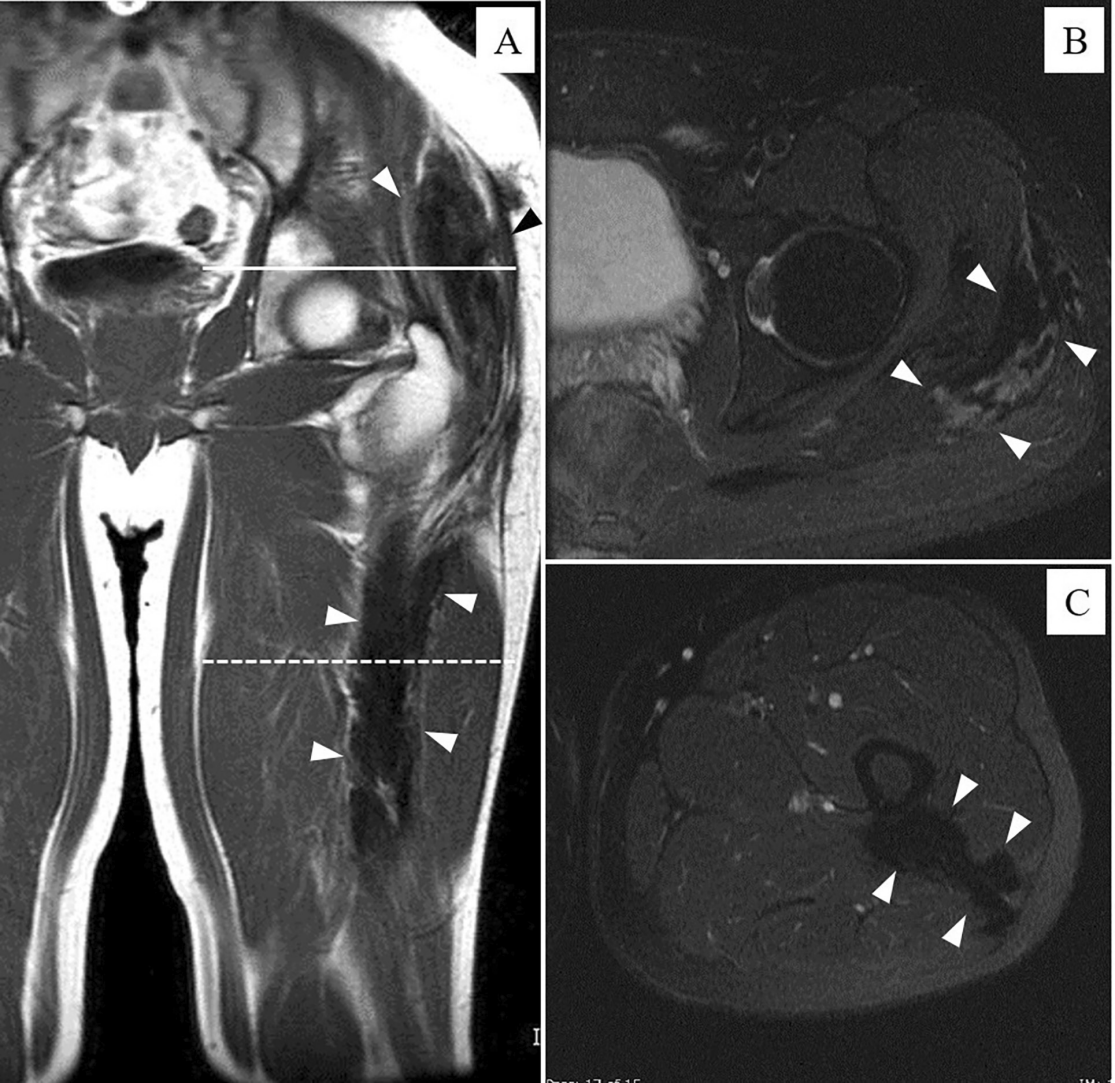
Magnetic resonance imaging analysis. Homogeneously low‐intensity lesion on the T1‐weighted and short TI inversion recovery images of the left gluteus maximus and gluteus medius muscles. The tumor shows increasing low signal area and edema of the muscle 3 years after CIRT. The tumor size decreased. The tumor is indicated by arrowheads. (A) T1WI coronal sequence (gluteus muscle slice, femoral muscle slice). (B) Short TI inversion recovery axial sequence (gluteus muscle slice). (C) Short TI inversion recovery axial sequence (femoral muscle slice).

A 31‐year‐old man presented at Sapporo Medical University Hospital in March 2011 with a 1‐year history of increased pain in the left gluteal region. No family or personal history of tumor was reported. There was no history of trauma or surgery. The patient was not taking any medications. Physical examination revealed tenderness of the left buttock, but without clear swelling or restriction of the range of motion of the hip. Magnetic resonance imaging (MRI) showed a homogeneously low‐intensity lesion on T1‐ and T2‐weighted images. It had a maximum diameter of 35 mm in the left gluteus maximus and gluteus medius muscles and extended to the vastus lateralis and biceps femoris (long head) (Figure 1). The lesion showed prominent Gd‐contrast enhancement at the point where we planned the biopsy. Needle biopsy revealed a fibrous tumor consisting of spindle‐shaped tumor cells with bland oval nuclei and a few small nucleoli (Figure 2A). The tumor had small vessels and an abundant fibrous matrix. Immunohistochemistry revealed nuclear expression of β‐catenin (Figure 2B), leading to the diagnosis of DF. The patient was initially observed under an “active surveillance” policy. Non‐surgical treatment, including a cyclooxygenase 2 inhibitor and tamoxifen, was administered because the patient had experienced increased pain for 8 years. However, the pain in his left thigh did not improve. In 2011, molecular‐targeted drugs such as sorafenib and imatinib were not available for use in Japan. The tumor was large, and surgical resection could lead to loss of function and decreased quality of life. Therefore, RT was considered after a thorough discussion with the patient in anticipation of pain relief. CIRT was considered likely to be effective and safer for a DF tumor because it can precisely irradiate the target lesions. The patient received 67.2 Gy (relative biological effectiveness) in 16 fractions for 4 weeks using the field patching technique at Gunma University Heavy Ion Medical Center. Figure 3 shows the CIRT plan for the patient. Although no tumor shrinkage was observed, the tumor showed an increasing low‐signal area 3 years after CIRT (Figure 4). The patient developed grade 1 edema and peripheral neuropathy (CTCAE Ver.5.0.) in the lower leg but the pain was relieved and required no medication. Thereafter, he did not visit the outpatient clinic for 5 years.

**FIGURE 5:**
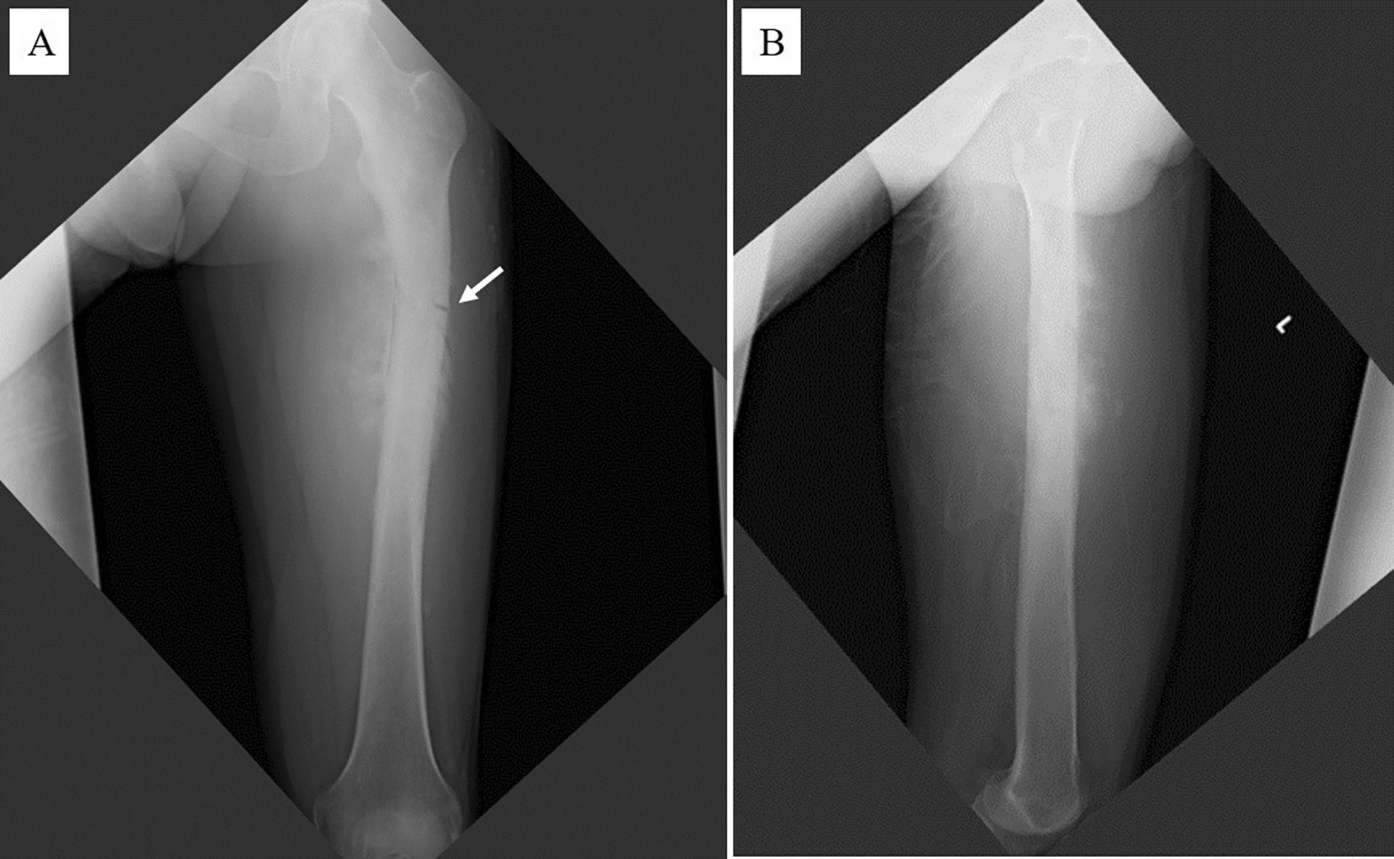
X‐ray obtained at 8 years after irradiation showing a bone destructive lesion with aggressive periosteal reaction and calcification and pathological fracture of the left femur. (A) Anteroposterior view of the left femur. (B) Lateral view of the left femur.

**FIGURE 6:**
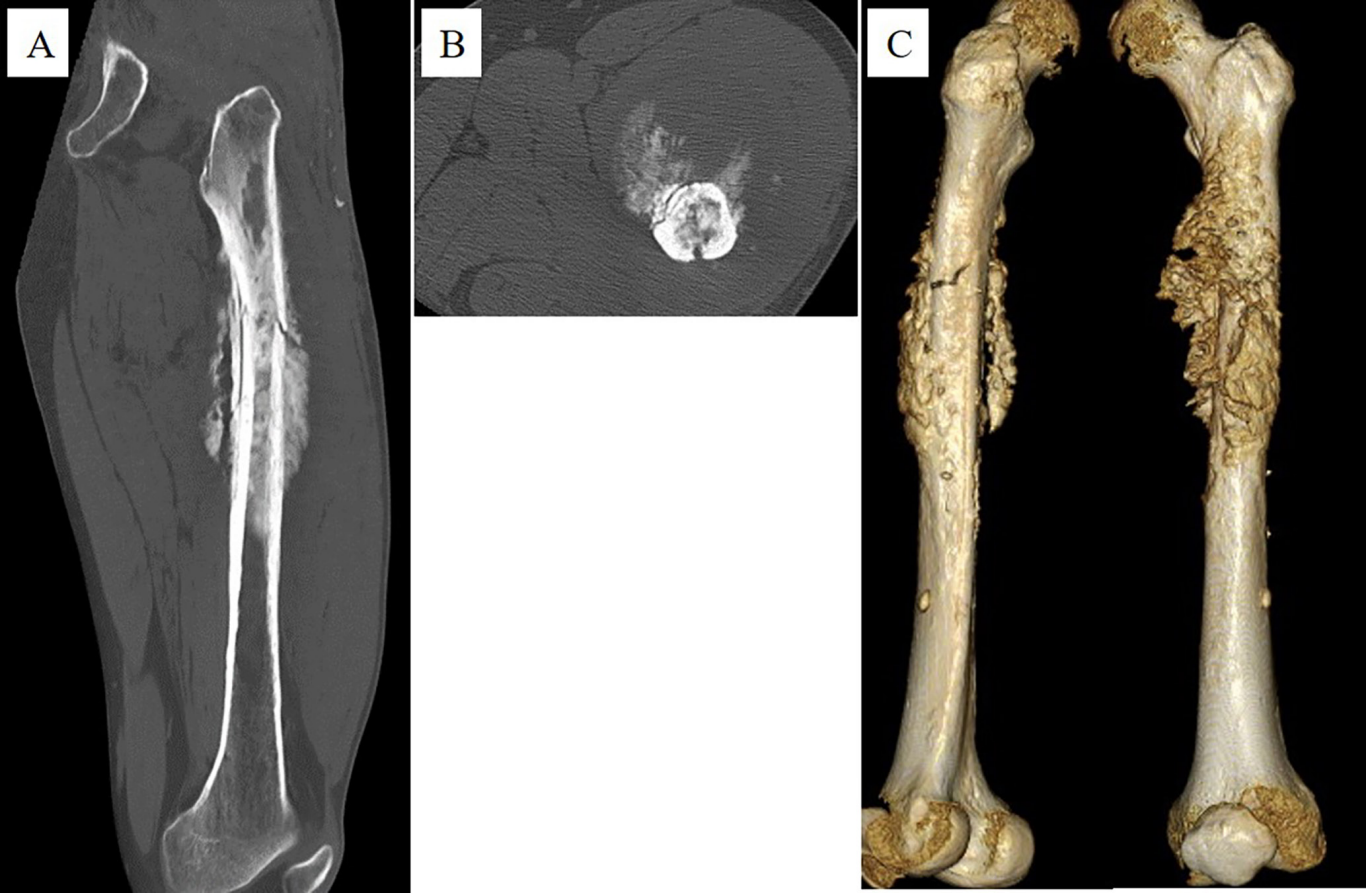
Computed tomography showing extra‐osseous tumor extension with ossifying tumor‐induced calcifications at 8 years after radiation. (A) Sagittal sequence. (B) Axial sequence. (C) Three‐dimensional CT.

**FIGURE 7:**
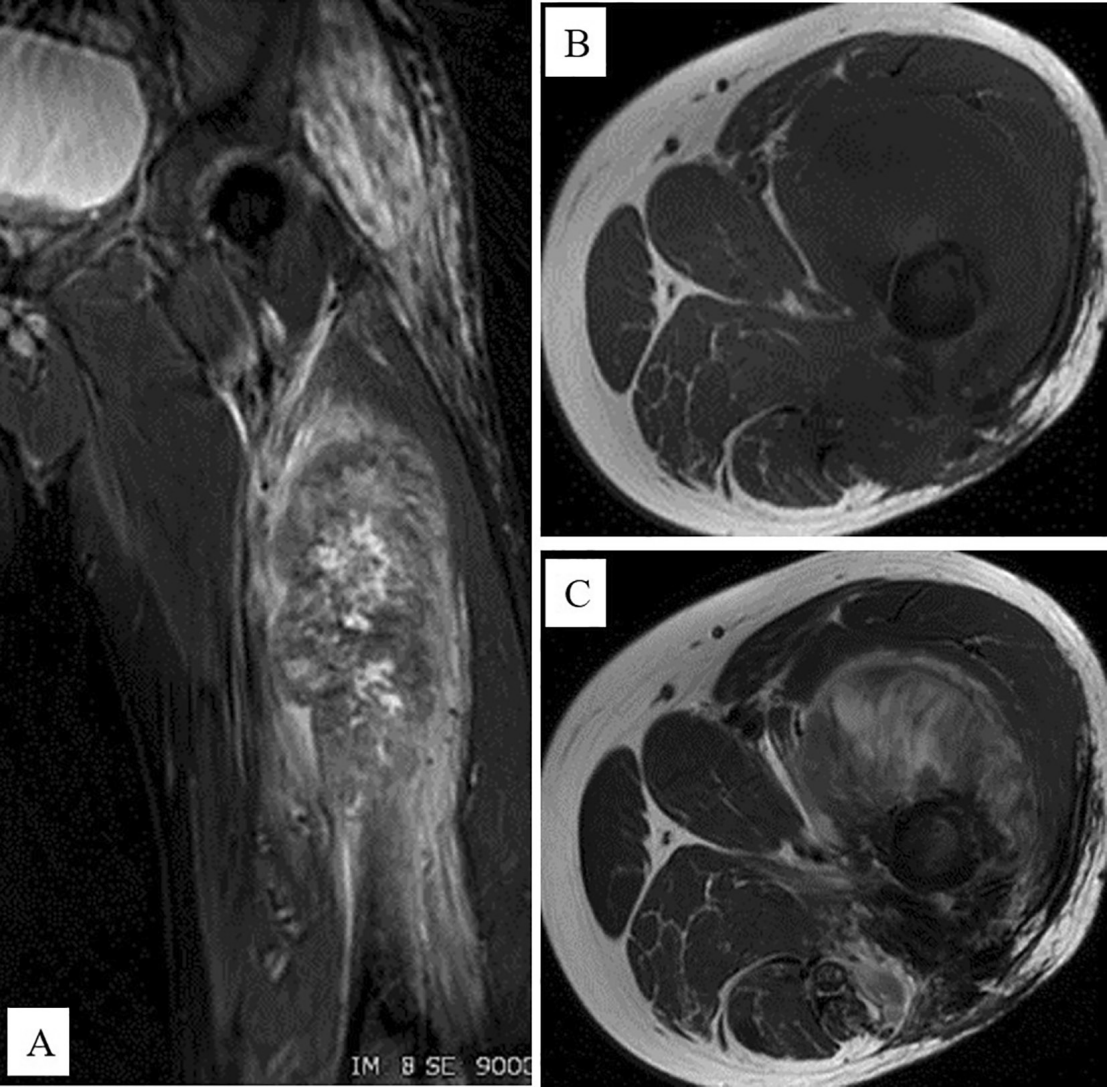
Magnetic resonance imaging analysis. Intramedullary tumor with low to intermediate and high signal on axial T2WI. Circumferential extra‐osseous tumor mostly shows a high signal, measuring 70 × 71 × 210 mm at 8 years after radiation. (A) Short TI inversion recovery coronal sequence. (B) T1WI axial sequence (femur muscle slice). (C) T2 WI axial sequence (femur muscle slice).

**FIGURE 8:**
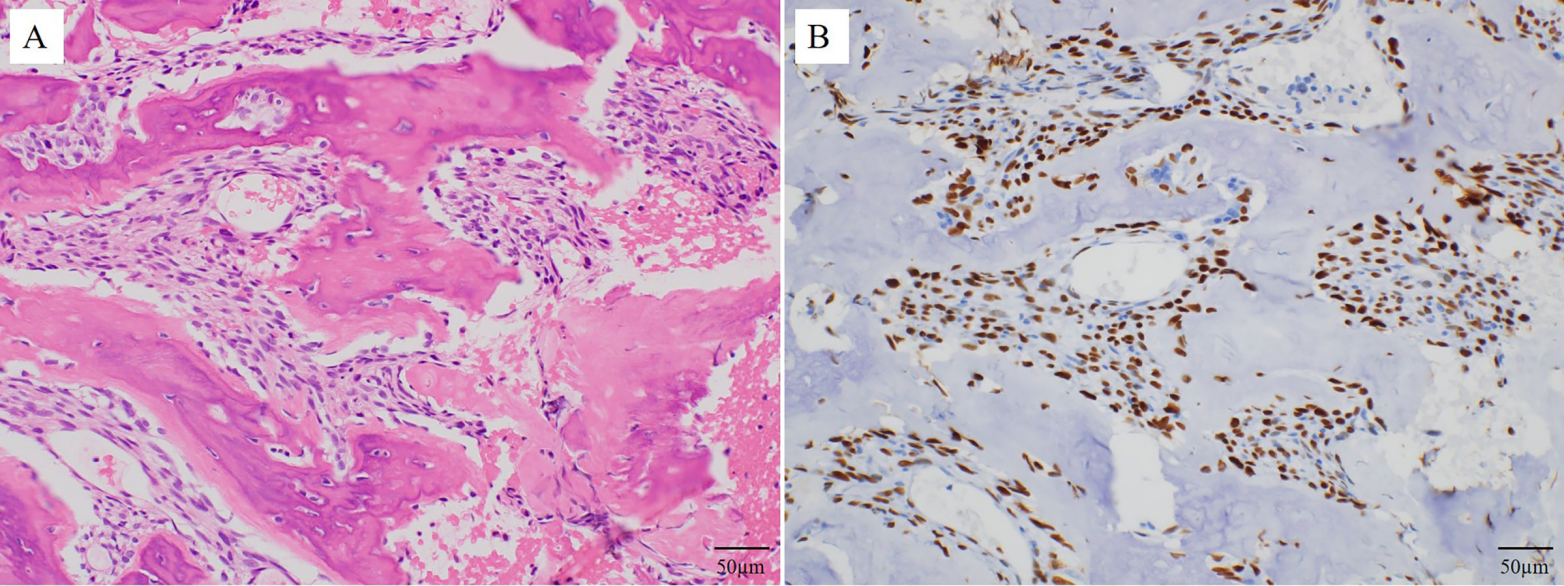
(A) The tumor consists of spindle to polygonal tumor cells with hyperchromatic nuclei. Osteoid and bone formation by the malignant cells is observed (Hematoxylin and eosin, ×200). Histopathological diagnosis was osteosarcoma. (B) Tumor cells are positive for Special AT‐rich sequence‐binding protein 2 (SATB2) on immunohistochemistry, × 200.

**FIGURE 9:**
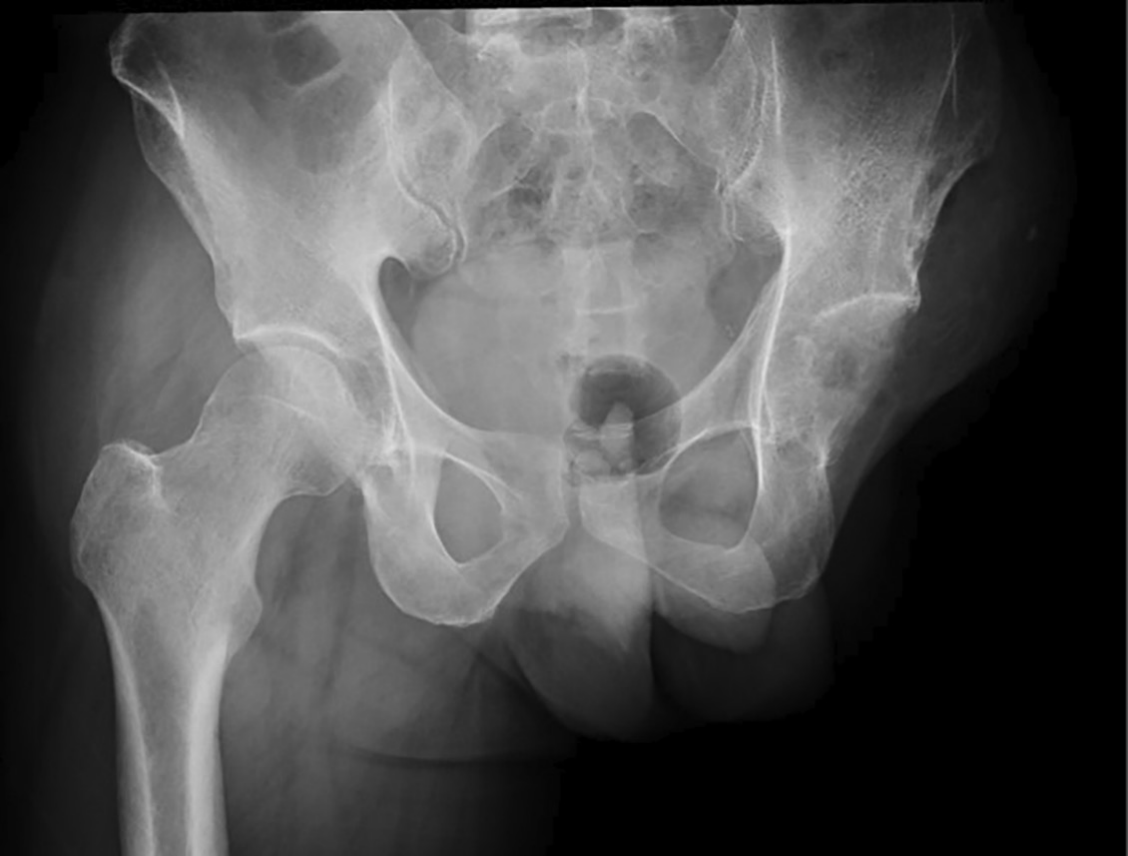
X‐ray after surgery with left hip disarticulation.

**FIGURE 10:**
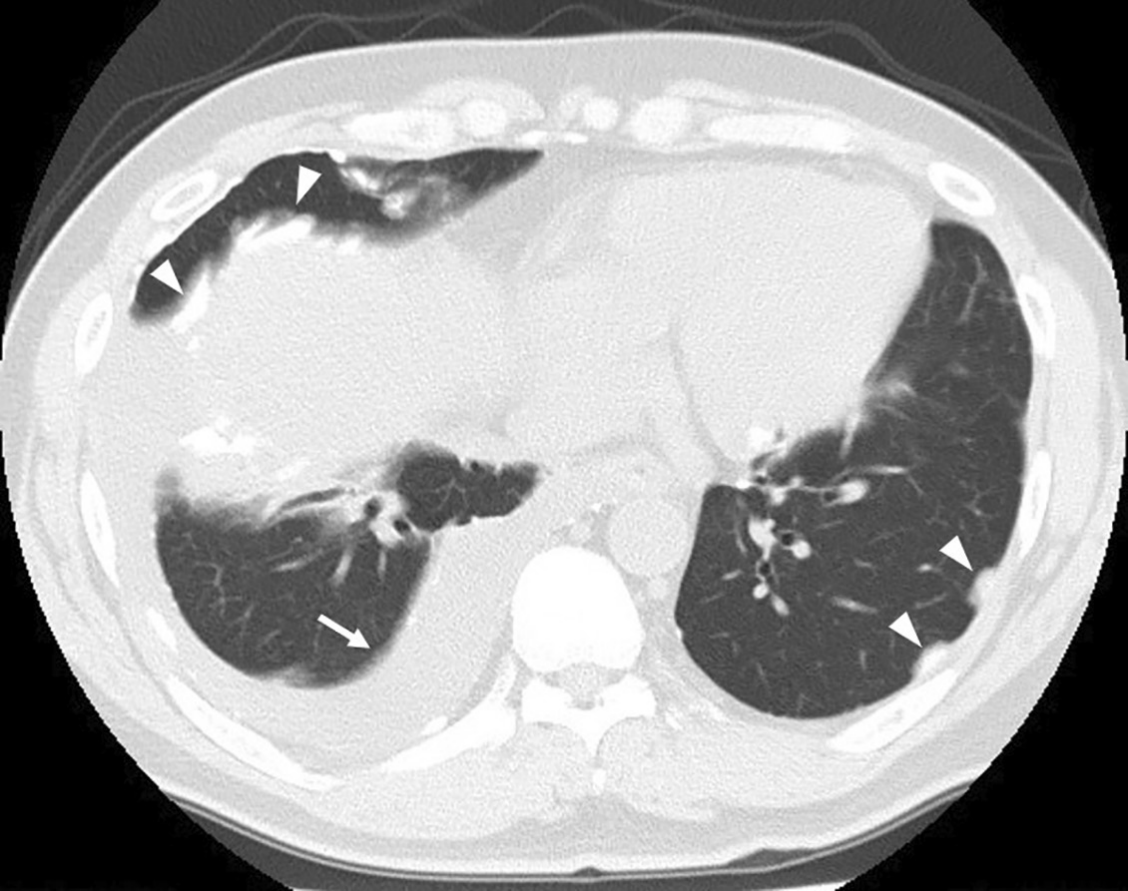
Computed tomography showing pleural dissemination (▲) and effusion (→).

In September 2020, 8 years after CIRT, the patient revisited Sapporo Medical University Hospital complaining of pain in the left thigh and inability to walk. Radiographs showed a periosteal reaction and calcification of the left femur (Figure 5). Pathological fractures were detected using CT. Extraosseous tumor extension with ossification of the tumor‐induced calcification was also evident (Figure 6). MRI showed that the tumor had spread from the bone marrow to the extraosseous soft tissue. The intramedullary tumors had showed low‐intermediate and high signals on axial T2 WI, whereas the circumferential extraosseous tumors mostly showed high signal. The tumor measured 70 × 71 × 210 mm (Figure 7). In the view of calcification and periosteal reaction, the main differential diagnoses include osteosarcoma, chronic sclerosing osteomyelitis, or metastatic prostate cancer. At revisit, the patient's blood test results were as follows: WBC 7400/μL (normal range, 3300–8600/μl) (69.5% neutrophils), CRP 0.33 mg/dL (normal range, 0.00–0.14/μl), ALP (IFCC) 132 U/L (normal range, 38–113 U/L), PSA 0.6 ng/mL (normal range, 0.00–3.53 ng/mL). Osteomyelitis and prostate cancer were therefore ruled out based on the above results. An open biopsy of the soft tissue was performed, and osteosarcoma was pathologically diagnosed. TNM staging was Stage IIB (T2N0M0) [5]. The biopsy specimen showed the proliferation of spindle‐to‐polygonal tumor cells with hyperchromatic nuclei. The tumor demonstrated osteoid formation (Figure 8A). Immunohistochemistry revealed that the tumor cells were positive for AT‐rich sequence‐binding protein 2 (SATB2) (Figure 8B). Therefore, we considered the lesion to be a postirradiation sarcoma, which was consistent with the criteria reported by Cahan et al. [6]. Because metastasis was not recognized, chemotherapy and surgery were performed. The patient received a chemotherapy regimen comprising cisplatin (CDDP), doxorubicin (DXR) and methotrexate (MTX) based on the Japanese Clinical Oncology Group (JCOG) protocol (i.e., two cycles of CDDP 100 mg/m^2^ on day 1; DXR 30 mg/m^2^ on days 1 and 2; and MTX 10 g/m^2^ on days 22 and 29 every 5 weeks as neo‐adjuvant chemotherapies, followed by a cycle of CDDP 100 mg/m^2^ on day 1 and DXR 30 mg/m^2^ on days 1 and 2 as an adjuvant chemotherapy after surgical treatment) [7]. He underwent en bloc excision of the tumor with left hip disarticulation (Figure 9). Pulmonary metastasis was detected 6 and 17 months after the definitive surgery. Because it was a single metastatic lesion, pulmonary metastasectomy was performed for both lesions. However, multiple lung metastases appeared 2 months after the second metastasectomy. The patient refused hospitalization and was treated with pazopanib, an oral drug that can be administered on an outpatient basis. The patient received pazopanib 600 mg/day for 4 months. However, the patient died 29 months after definitive surgery due to recurrence of multiple lung metastases (Figure 10).

## 3 Discussion

We reported for the first time that osteosarcoma developed during CIRT treatment for DF. The osteosarcoma had spread extraosseously to the anterior femur and was thought to have originated from the anterior femur. The CIRT treatment plan indicated that the anterior femur was irradiated with low‐dose radiation; therefore, we speculated that low‐dose CIRT may have been a factor in the development of osteosarcoma.

DF is a locally aggressive clonal fibroblastic proliferation characterized by often unpredictable clinical course. Pain is a common symptom in patients with DF and has a significant impact on the patient's quality of life. DF tumors (> 50 mm) are significantly associated with increased pain [8]. Surgery is a treatment modality, but the published recurrence rates are high (20%–70%) [9]. Spontaneous regression occurs in 20% of cases. Therefore, a paradigm shift toward more conservative management, which is active surveillance, has been introduced [10]. However, conservative management may not be appropriate, and intervention is usually recommended for patients with poorly controlled symptoms such as severe pain or disease progression [2].

RT is considered alternative local therapy and is indicated for extraperitoneal unresectable DF or when surgery for resectable DF can cause excessive morbidity [9]. A systematic literature review showed that patients with DF who received only RT had local control rates ranging from 65% to 83% [10]. While local control after RT is acceptable for patients aged ≤ 30 years, its associated high local control rate may be considered inferior to the outcomes of conservative treatment, such as initial observation [11]. The radiation damage caused by carbon‐ion beams is two to three times higher than that of the photon radiation method [12]. Compared to photon therapy, CIRT is not associated with oxygen effects or sublethal damage repair and has lower cell cycle‐related radiosensitivity. These properties provide a rationale for the initial application of carbon ions in the treatment of radioresistant and/or hypoxic diseases. Additionally, there are indications that the sharp dose distribution allows delivery of therapeutic doses to critical radiation‐sensitive organs (in this case, the bone marrow tissue of the femur) and adjacent disease [13, 14, 15]. Therefore, CIRT, as opposed to photon therapy, has the advantage of reducing some toxicities associated with RT, including secondary malignancies. There is no direct comparison of the risk of second malignancies associated with CIRT and photon therapy. However, a study examining the risk of second malignancies in patients with prostate cancer found lower risks associated with CIRT than with photon radiation. This is likely due to reduced exposure of normal tissue to CIRT outside the target volume [16]. There are very few case reports on the effectiveness of CIRT for the treatment of DF. Seidensaal et al. reported the RT treatment outcomes for aggressive fibromatosis in a single institution, and only one patient was treated with CIRT [17]. Of 44 patients who underwent RT treatment for DF, 15 received radiation of 60 Gy or more. Nagata et al. reported a case of DF without recurrence in the abdominal wall 3 years after CIRT [18]. In our case, the irradiation field was as large as 30 cm; therefore, we used a field‐patching technique [19].

A systematic review of secondary malignancies caused by RT showed that the period between primary treatment and second primary malignancy ranged from 1 to 37 years [20]. The patient in this case developed osteosarcoma 8 years after CIRT. We diagnosed the patient with radiation‐induced osteosarcoma based on the following criteria [6]; (1) history of RT, (2) asymptomatic latency period of several years, (3) occurrence of sarcoma within a previously irradiated field, and (4) histological confirmation of the sarcomatous nature of the post‐irradiation lesion.

The osteosarcoma had spread extraosseously to the anterior femur and was believed to have originated from the anterior femur. The CIRT treatment plan indicated that the anterior femur was irradiated with low‐dose radiation; thus, low‐dose RT may have contributed to osteosarcoma development. In tissues exposed to low doses, the linear non‐threshold of the dose–response curve means that a single electron can induce a DNA double‐strand break, which, although unlikely, may cause an early carcinogenic event [21]. At low doses, non‐target effects can increase the size of the susceptible target from a single cell to an entire tissue or part of a tissue, thereby increasing cancer risk. Protective mechanisms have also been hypothesized wherein damaged cells are removed from the organism by intercellular signaling, thereby protecting tissue stability [22].

## 4 Conclusion

The low radiation dose to the femur may have caused secondary malignancy.

## Author Contributions

**Mizuki Aketo:** conceptualization, data curation, investigation, writing – original draft. **Makoto Emori:** conceptualization, investigation, writing – original draft. **Kohichi Takada:** conceptualization, resources, data curation. **Kazuyuki Murase:** investigation, software, validation. **Yohei Arihara:** investigation, software, validation. **Junya Shimizu:** investigation, software, validation. **Yasutaka Murahashi:** investigation, software, validation. **Masahiko Okamoto:** investigation, software, validation. **Shintaro Sugita:** investigation, software, validation. **Atsushi Teramoto:** conceptualization, data curation, writing – original draft, writing – review and editing, resources.

## Ethics Statement

All procedures performed in this study involving human participants were in accordance with the ethical standards of the institutional and/or national research committee and with the 1964 Helsinki declaration and its later amendments or comparable ethical standards.

## Consent

Written informed consent was obtained from the patient's mother for publication of this case report and any accompanying images.

## Conflicts of Interest

The authors declare no conflicts of interest.

## Data Availability

The data that support the findings of this study are available from the corresponding author upon reasonable request.
